# Deceptive clinical course of mucinous ovarian carcinoma mimicking pelvic abscess in a postmenopausal woman: An exceptional case report

**DOI:** 10.18632/oncoscience.650

**Published:** 2026-03-11

**Authors:** Aparna Jarathi, Sunitha Geddada, Chandramouli Ramalingam, Naina Kumar, Anusha Devalla, Ashwini Pitambra, Ashutosh Rath, NagaSai Divya Kari, Uday Reddy Janke, B Surender Reddy, Ajay Kumar Kondeti

**Affiliations:** ^1^Department of Obstetrics and Gynaecology, All India Institute of Medical Sciences (AIIMS), Bibinagar, Hyderabad, India; ^2^Department of Radiation Oncology, AIIMS, Bibinagar, Hyderabad, India; ^3^Department of Pathology and Lab Medicine, AIIMS, Bibinagar, Hyderabad, India

**Keywords:** mucinous cystadenocarcinoma, pelvic abscess, carcinoma ovary, histopathological diagnosis

## Abstract

Background: Mucinous ovarian carcinoma (MOC) is a rare subtype of epithelial ovarian cancer (3–5%), typically affecting women between 20 and 40 years old. It often presents diagnostic and management challenges.

Case Report: We present an exceptional case of a postmenopausal woman with abdominal pain disguised as a pelvic abscess. A 73-year-old postmenopausal woman presented with abdominal pain for 6 months and postmenopausal spotting for 1 month. Initial imaging (CT scan) suggested a pelvic abscess, which was managed conservatively. However, persistent symptoms and a complex adnexal mass prompted further evaluation. MRI revealed a multiloculated right adnexal mass, and tumour markers (CA-125, CEA, HE4) were elevated. Surgical staging included total abdominal hysterectomy, bilateral salpingo-oophorectomy, and omentectomy. Histopathology confirmed stage IC2 mucinous adenocarcinoma. Postoperative recovery was uneventful, and the patient received six cycles of carboplatin and paclitaxel as adjuvant chemotherapy.

Conclusions: This case emphasizes the diagnostic challenge of MOC in postmenopausal women.

Misinterpretation as benign pelvic pathology can delay appropriate treatment. Precise imaging, tumour markers, and a multidisciplinary approach are critical for early diagnosis and improved outcomes.

## INTRODUCTION

Mucinous ovarian carcinoma (MOC) is an uncommon histological subtype of epithelial ovarian cancer, accounting for approximately 3–5% of all ovarian malignancies [1]. It is characterized by mucin-producing epithelial cells and tends to present as a large, unilateral, multiloculated cystic mass. Mucins have also been found to be associated with chemoresistance in mucinous carcinomas [2]. While MOC typically affects women in the reproductive age group, most commonly between 20 and 40 years, it is rare in postmenopausal women, often leading to diagnostic challenges [3, 4].

The clinical presentation of MOC can be nonspecific, ranging from abdominal discomfort and bloating to gastrointestinal symptoms and pelvic pressure. In postmenopausal women, these subtle symptoms can overlap with other benign gynecologic or gastrointestinal conditions, such as diverticulitis, tubo-ovarian abscess, or pelvic inflammatory disease. Furthermore, radiological findings may not be definitive, especially when imaging reveals complex adnexal masses with features mimicking inflammatory or infectious processes [5].

Pelvic abscess is an uncommon diagnosis in elderly, postmenopausal women without classical risk factors such as recent instrumentation, intrauterine device use, or active infection.

Misdiagnosis can result in delays in definitive surgical management and staging. Tumor markers such as CA-125, carcinoembryonic antigen (CEA), and human epididymis protein 4 (HE4), although nonspecific, may aid in raising suspicion of malignancy when interpreted in the appropriate clinical context [6, 7].

In this report, we present a diagnostically deceptive case of mucinous ovarian carcinoma in a 73 year-old postmenopausal woman initially misdiagnosed as a pelvic abscess.

## CASE SUMMARY

A 73-year-old multiparous (P5L5) postmenopausal woman presented to the Gynaecology outpatient clinic with a 6-month history of dull, aching abdominal pain, followed by progressive abdominal distension and postmenopausal bleeding for the past 2 months. Her medical, surgical, and family histories were unremarkable for malignancy.

**Figure 1:**
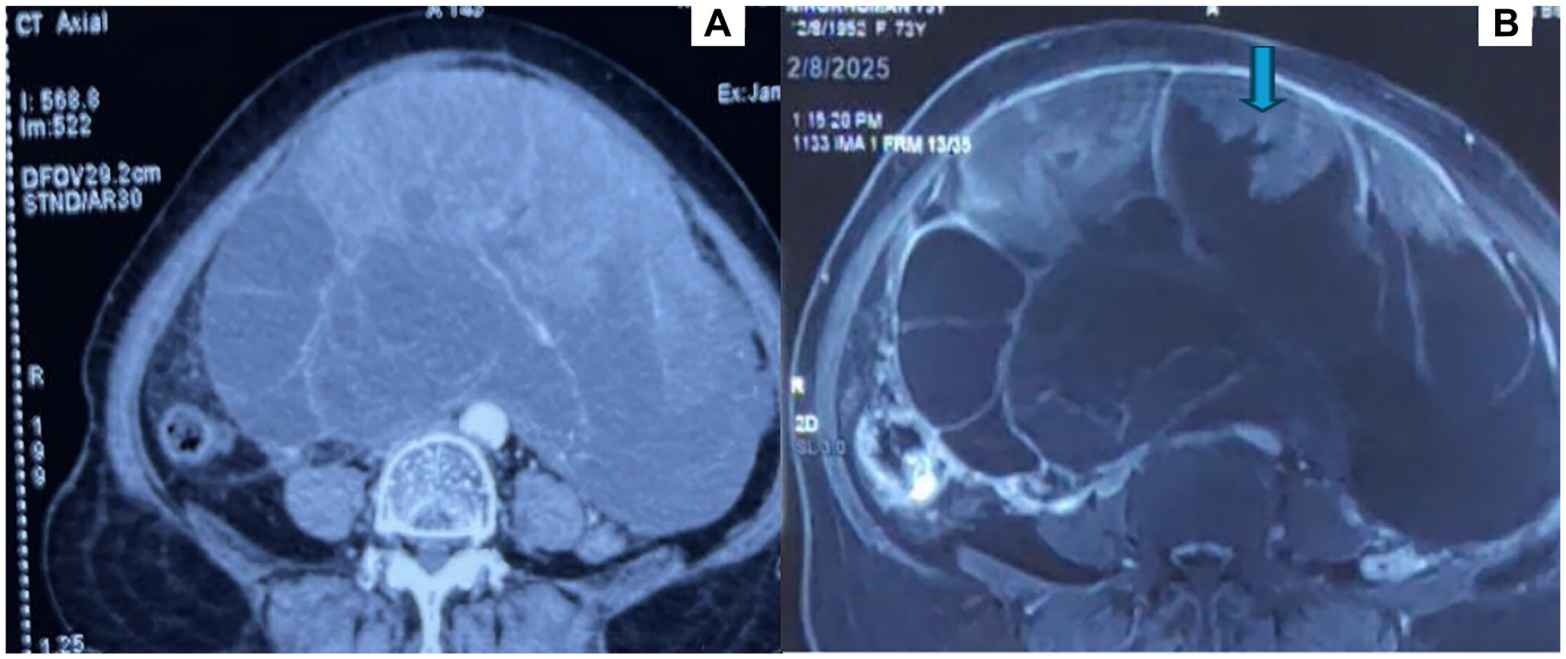
Radiological imaging of the abdominal lesion. (**A**) CT axial view showing a large, lobulated, multiloculated solid cystic lesion in abdomen. (**B**) MRI T2 Image showing a large, lobulated, multiloculated solid cystic lesion in abdomen with hyperintense solid components (arrow).

Six months earlier, she underwent an evaluation of abdominal pain in her hometown. Initial ultrasonography and CT scan revealed a right adnexal lesion: a well-defined, multiloculated cystic mass with air foci and septations (12 × 14 cm), provisionally diagnosed as a pelvic abscess (Figure 1A). She was advised by conservative management at that time.

Later, at an oncology center, she was advised evaluation for abdominal distension and bleeding, which included Pap smear (atrophic, negative for epithelial malignancy), endometrial biopsy (simple hyperplasia without atypia), and an ultrasound guided biopsy of the adnexal mass (suggestive of spindle cell lesion). Tumor markers were elevated: serum CA-125: 374 U/ml and serum CEA: 292 ng/ml. Colonoscopy was normal. A whole-body PET-CT scan demonstrated a primary pelvic malignancy with no other active lesions.

On general examination, she appeared cachectic and pale. Abdominal examination revealed a 22 × 18 cm mass arising from the pelvis, extending to the upper abdomen, with a solid-cystic consistency, restricted mobility, and no tenderness. Pelvic examination revealed a hypertrophied cervix and an adnexal mass filling the abdomen. MRI pelvis demonstrated a large, multiloculated solid-cystic lesion (24 × 16 × 27 cm) arising from the right adnexa, extending up to the renal hilum. Solid components were T2 hyperintense with diffusion restriction. There were no peritoneal or para-aortic deposits, no significant lymphadenopathy, and only mild ascites (Figure 1B).

**Figure 2:**
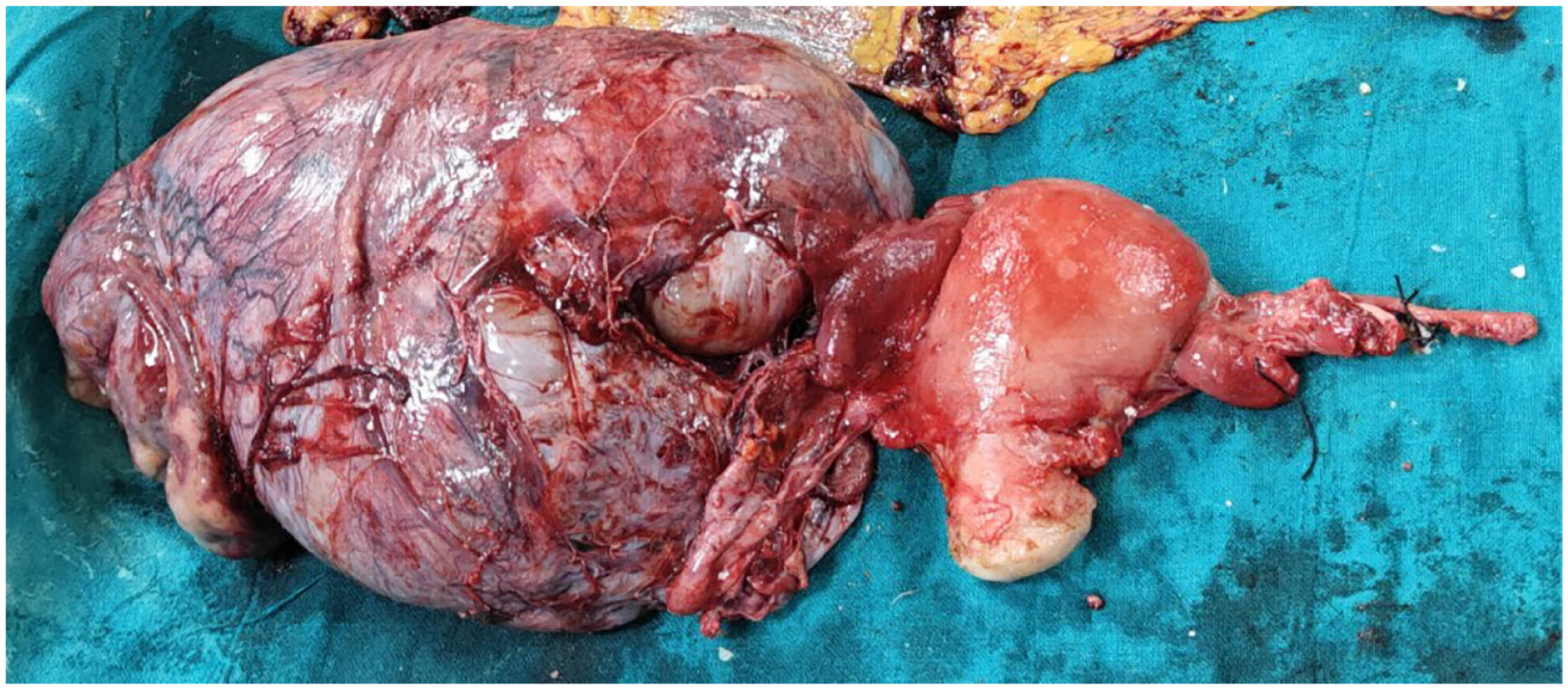
Post-surgical specimen showing a right complex solid cystic ovarian mass with a normal-sized uterus, left atrophic ovary.

Staging laparotomy was performed initially. Intraoperatively, mild ascites was noted. The right ovary was grossly enlarged with solid and cystic components, densely adherent to the colon. A capsular breach (1 × 2 cm) released foul-smelling purulent fluid mixed with mucinous material. Eventually, a Total abdominal hysterectomy with bilateral salpingo-oophorectomy, bilateral pelvic lymphadenectomy, and infracolic omentectomy was performed. Gross inspection revealed no peritoneal or omental deposits (Figure 2).

**Figure 3:**
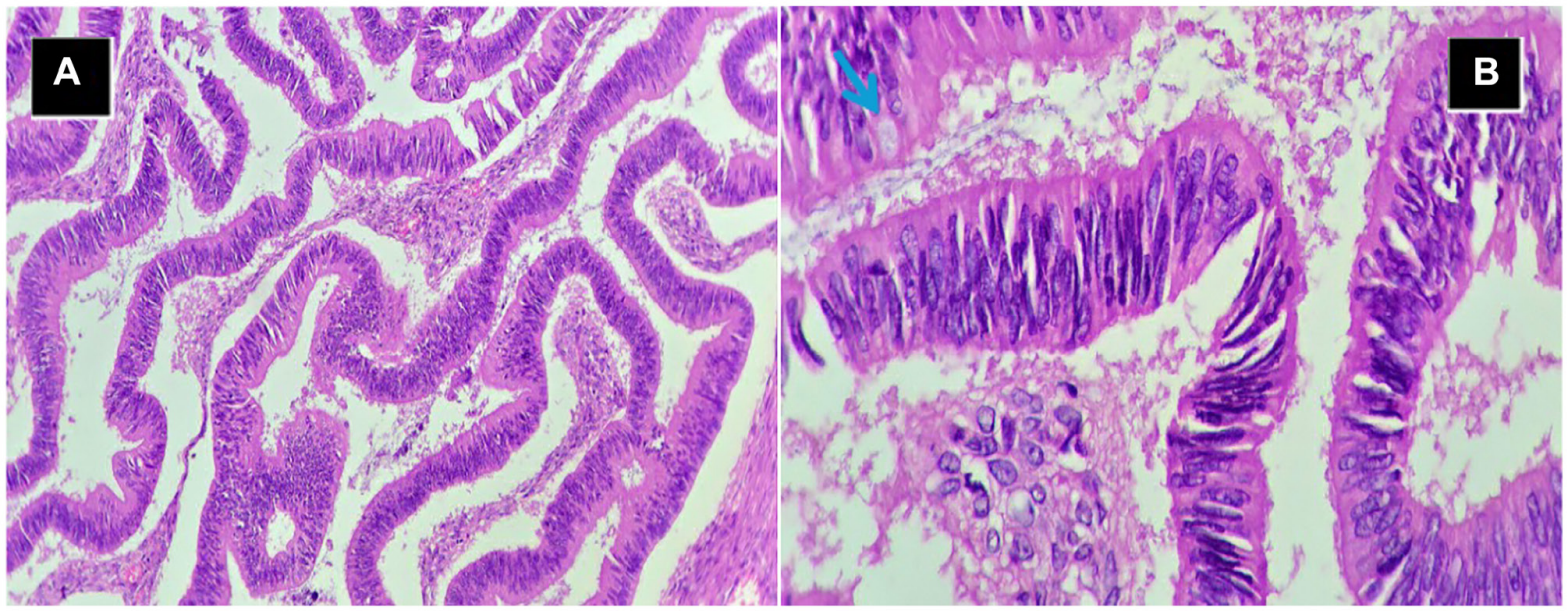
Tumor histopathology. (**A**) H&E 10X- Tumor lined by glands showing Intestinal-type differentiation with Villoglandular architecture infiltrating ovarian stroma. (**B**) H&E 40X- Intestinal-type epithelium with interspersed goblet celt (blue arrow).

Histopathology confirmed mucinous adenocarcinoma of the right ovary, FIGO stage IC2 –TNM stage pT1C2N0 (Figure 3). Ascitic fluid cytology was negative, with no pelvic lymph node or omental involvement.

Postoperatively, the patient developed persistent pus-like vaginal discharge from day 5. Culture grew extended-spectrum β-lactamase (ESBL)-producing *Escherichia coli*. She responded to culture-sensitive intravenous antibiotics for over 2 weeks and was subsequently discharged. The patient received six cycles of adjuvant carboplatin and paclitaxel chemotherapy in the radiation oncology department and remains under regular follow-up.

## DISCUSSION

Mucinous ovarian carcinoma (MOC) is an uncommon subtype of epithelial ovarian cancer, accounting for only 3–5% of cases [1, 8]. It is typically seen in younger, reproductive-age women, making its occurrence in postmenopausal patients rare and diagnostically challenging [9]. The present case demonstrates the deceptive clinical course of MOC in a 73-year-old woman, where the tumor initially mimicked a pelvic abscess.

Such presentations may result in misinterpretation, delayed diagnosis, and potentially suboptimal management. The key differential diagnoses that may mimic MOC in postmenopausal women, including pelvic abscess, tubo-ovarian abscess, diverticular abscess, metastatic gastrointestinal malignancy, and other complex adnexal masses, emphasizing overlapping clinical and radiological features [9].

In postmenopausal women, adnexal masses warrant careful evaluation with a high index of suspicion for malignancy. MOC in postmenopausal women is particularly significant, given its rarity, frequent misdiagnosis, large tumor size at presentation, and the risk of delayed definitive surgical management [10]. Radiological features of MOC often overlap with those of benign conditions, including tubo-ovarian abscesses, diverticular abscesses, or other inflammatory pelvic processes [11].

In our case, initial CT imaging revealed a multiloculated cystic lesion with septations and air foci, which was interpreted as a pelvic abscess. While abscesses are uncommon in elderly women without risk factors such as recent instrumentation or immunosuppression, the initial conservative approach delayed surgical exploration. This highlights the importance of integrating radiological findings with clinical context and tumor marker assessment.

MRI plays a critical role in refining the diagnosis of complex adnexal masses [12]. According to ESUR guidelines, MRI is superior to CT in characterizing indeterminate lesions. In this case, MRI demonstrated a multiloculated solid-cystic right adnexal mass with diffusion restriction, findings highly suggestive of malignancy [5]. Alongside imaging, serum biomarkers aid in distinguishing between benign and malignant pelvic pathology. Elevated CA-125 and CEA levels in this patient raised further suspicion of carcinoma. Previous studies have shown that combining multiple markers, including HE4, improves diagnostic sensitivity and specificity [13].

Surgical staging remains the cornerstone of management for MOC [14]. In our patient, staging laparotomy revealed a grossly enlarged ovary with capsular rupture and mucinous spillage, leading to a FIGO Stage IC2 diagnosis. Complete staging with hysterectomy, bilateral salpingooophorectomy, lymphadenectomy, and omentectomy is essential, both for diagnosis and for determining prognosis. Although the role of adjuvant chemotherapy in early-stage MOC remains debated due to limited chemosensitivity compared with high-grade serous carcinoma, platinumbased regimens such as carboplatin and paclitaxel are recommended for stage IC disease or when adverse features are present [15]. Our patient completed six cycles of adjuvant chemotherapy uneventfully.

A unique aspect of this case was the postoperative infectious complication. The presence of foulsmelling purulent fluid intraoperatively led to persistent vaginal discharge, with cultures growing ESBL-producing E. coli. Such infections may complicate recovery, particularly when tumor rupture mimics abscess formation [16]. Nevertheless, culture-directed antibiotics achieved resolution, allowing the patient to proceed with adjuvant therapy.

This case underscores three key learning points. First, adnexal masses in postmenopausal women should always raise concern for malignancy, even when imaging suggests an abscess. Second,

MRI and tumor markers provide valuable diagnostic clarity when CT findings are inconclusive. Third, a multidisciplinary approach involving gynecologic oncology, radiology, pathology, and infectious disease specialists ensures timely diagnosis, optimal treatment, and effective management of complications.

## CONCLUSIONS

Mucinous ovarian carcinoma (MOC) can present deceptively, especially in postmenopausal women, mimicking benign conditions such as pelvic abscesses. MRI is superior to CT in characterising complex adnexal masses and should be used when initial imaging is inconclusive or clinical suspicion remains. Tumor markers (CA-125, CEA, HE4) can help distinguish between benign and malignant pelvic masses and should be evaluated in persistent adnexal pathology.

Early surgical intervention and complete staging are essential in achieving a definitive diagnosis and initiating appropriate oncologic management. A multidisciplinary approach, involving gynecologic oncology, radiology, and pathology, improves diagnostic accuracy.
